# Evaluation of mucosal-associated invariant T-cells as a potential biomarker to predict infection risk in liver cirrhosis

**DOI:** 10.1371/journal.pone.0294695

**Published:** 2024-05-01

**Authors:** Bonnie Bengtsson, Christopher Maucourant, Johan K. Sandberg, Niklas K. Björkström, Hannes Hagström

**Affiliations:** 1 Department of Internal Medicine, Section of Gastroenterology, Södersjukhuset, Stockholm, Sweden; 2 Unit of Gastroenterology and Rheumatology, Department of Medicine Huddinge, Karolinska Institutet, Stockholm, Sweden; 3 Center for Infectious Medicine, Department of Medicine Huddinge, Karolinska Institutet, Stockholm, Sweden; Medical University of Graz: Medizinische Universitat Graz, AUSTRIA

## Abstract

**Background and aims:**

Infection is a serious complication in patients with cirrhosis. Mucosal-associated invariant T (MAIT) cells are involved in the immune defense against infections and known to be impaired in several chronic conditions, including cirrhosis. Here, we evaluated if MAIT cell levels in peripheral blood are associated with risk of bacterial infections in patients with cirrhosis.

**Methods:**

Patients with cirrhosis seen at the Karolinska University Hospital, Stockholm, Sweden, between 2016 and 2019 were included. Levels of MAIT cells in peripheral blood were determined using flow cytometry. Baseline and follow-up data after at least two years of follow-up were collected by chart review for the primary outcome (bacterial infection) and secondary outcomes (decompensation and death). Competing risk and Cox regression were performed.

**Results:**

We included 106 patients with cirrhosis. The median MAIT cells fraction in the circulation was 0.8% in cirrhosis compared to 6.1% in healthy controls. In contrast to our hypothesis, we found an association in the adjusted analysis between relatively preserved MAIT cell levels, and a slightly higher risk to develop bacterial infections (adjusted subdistribution hazard ratio (aSHR) 1.15 (95%CI = 1.01–1.31). However, MAIT cell levels were not associated with the risk of hepatic decompensation (aSHR 1.19 (95%CI = 0.91–1.56)) nor with death (adjusted hazard ratio 1.10 (95%CI = 0.97–1.22)).

**Conclusions:**

Relatively preserved MAIT cell levels in blood of patients with cirrhosis were associated with a somewhat higher risk of bacterial infections. The clinical relevance of this might not be strong. MAIT cells might however be an interesting biomarker to explore in future studies.

## Introduction

Liver cirrhosis is considered an immunocompromised condition [1]. One reason for this is the systemic inflammation that occurs in cirrhosis, another is the reduced synthesis of liver-derived proteins important for the immune system [2]. The impaired antimicrobial surveillance function by the liver also contributes to an increased risk of infections as well as the dysregulated intestinal bacterial translocation that occurs, especially, in advances stages of cirrhosis [3, 4].

Bacterial infections are a common reason for developing decompensating liver events such as variceal bleeding and hepatic encephalopathy, and approximately 30% of the mortality seen in cirrhosis is attributable to infections [5]. Infections are present at admission or develop during hospital stay in 25–35% of patients with cirrhosis, which is 4–5 times higher than for other patients [3]. Urinary and respiratory tract infections are the most common sites of infections in cirrhosis [6]. Early recognition of bacterial infections is of high importance. Unfortunately, bacterial infection in cirrhosis commonly has an atypical clinical presentation and is therefore often diagnosed late [7].

Mucosal-associated invariant T-cells (MAIT cells) are unconventional T-cells with both adaptive and innate immune defense characteristics. They are defined by a semi-invariant T-cell receptor that recognizes bacterial metabolites presented on the MR-1 molecule [8]. MAIT cells are enriched in the intestinal mucosa and abundant in liver tissue where they represent 10–40% of liver T-cells [9, 10]. MAIT cells respond to a range of microbes and play an important role in the immune defense against infections [11–13].

Decreased levels of MAIT cells in blood have been described in several chronic liver diseases [14–17], but little is known about MAIT cell levels and impact on clinically relevant outcomes in these patients. We hypothesized that the degree of MAIT cell loss is associated with the risk for bacterial infections in patients with liver cirrhosis and may therefore serve as a predictive biomarker for infection risk in this population.

## Material and methods

### Study population

**Fig 1:**
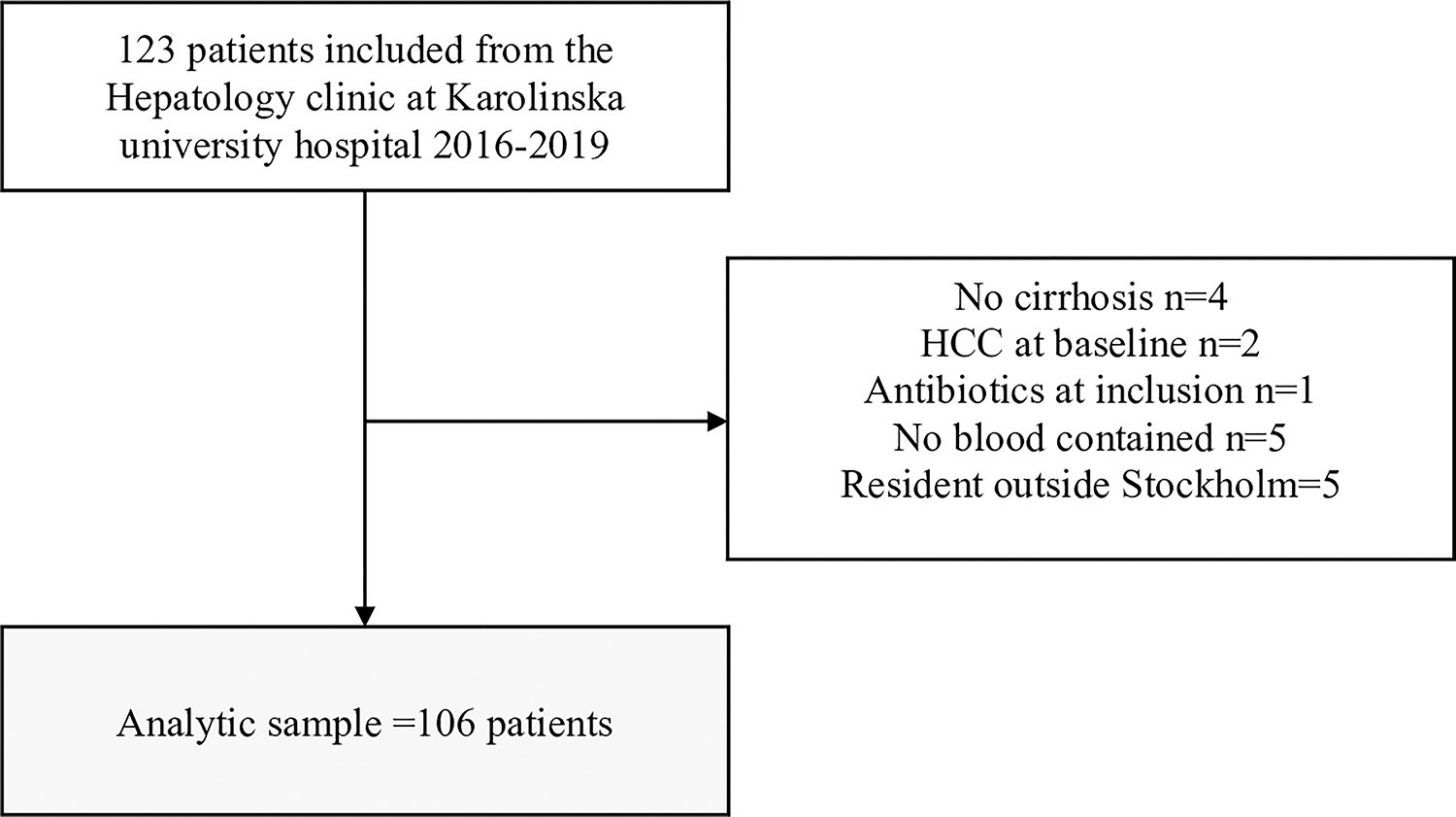
Flowchart for participant inclusion and exclusion.

Individuals with liver cirrhosis attending the Hepatology clinic at Karolinska University Hospital, Stockholm, Sweden, from 1 November 2016 until the 16 September 2019 were asked for participation. Exclusion criteria were active bacterial infections or treatment with antibiotics, except Rifaximin, at the time of inclusion. Patients with hepatocellular carcinoma (HCC) or other cancers were also excluded along with individuals with coinfection with HIV and hepatitis C. A flow chart describing the inclusion process is presented in Fig 1. A blood sample was drawn from the participant at the time of inclusion. Blood samples from 35 healthy controls matched on sex and age to serve as normal MAIT cell comparators were also obtained from anonymous blood donors from Stockholm, Sweden in October 2020.

#### Variables

Cirrhosis diagnosis and aetiology of liver disease were established according to standard diagnostic guidelines by a hepatologist. The primary outcome (bacterial infection) was defined as a bacterial infection that was diagnosed in a hospital (outpatient visit) or during hospitalization, and that required antibiotic treatment. The attending physician decided what tests and work-up that was suitable in each patient. The type of infection was defined as either: 1) spontaneous bacterial peritonitis (defined by ascitic fluid neutrophilic count >0.25x10^9^/l); 2) urinary tract infection (requiring a positive culture); 3) respiratory tract infection (chest x-ray consistent with pneumonia in combination with typical symptoms; 4) bacteremia (defined as a positive blood culture without a source of infection identified); and 5) others (clinically relevant such as endocarditis, wound infection, meningitis, and gastrointestinal tract infection, a positive culture from the affected site of infection was required). A wider definition of infection including bacterial *and* viral infections diagnosed in hospital *and* primary care was used in a sensitivity analysis.

Secondary outcomes were decompensation events (ascites, overt encephalopathy, and bleeding esophageal varices) in patients free of decompensation at baseline, and overall mortality (definitions are listed in S1 File).

#### Storage of blood and flow cytometry

**Fig 2:**
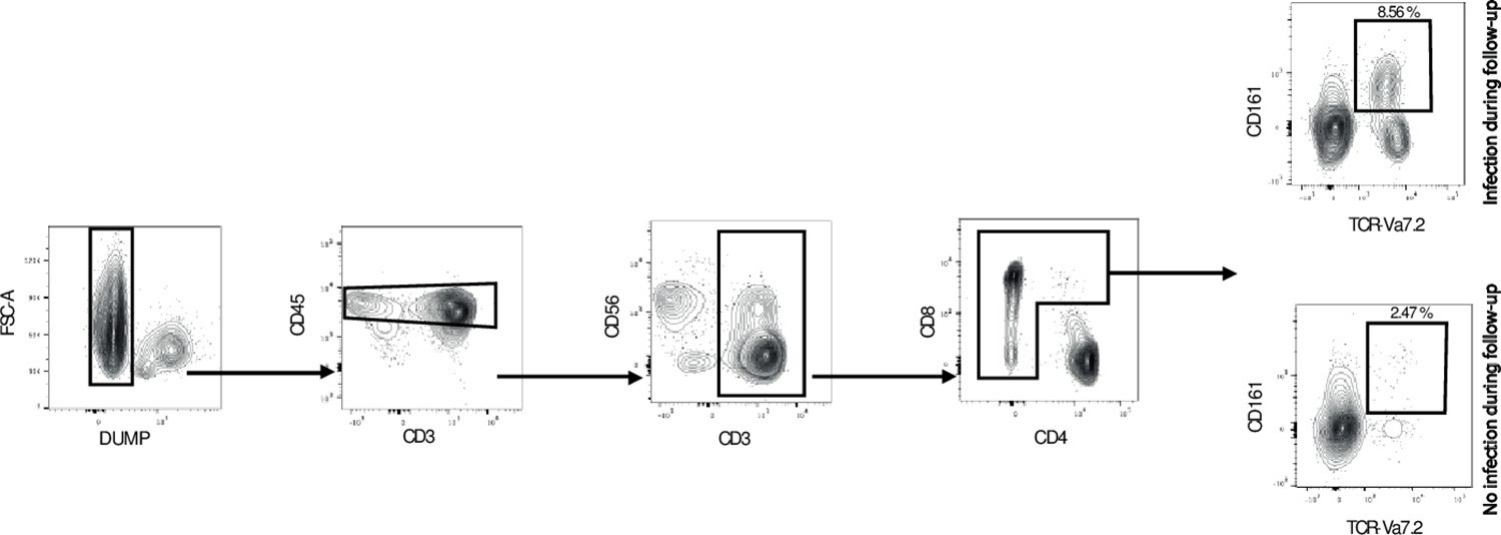
MAIT cells frequency in patients developing infection or not. (A) Gating scheme used to identify MAIT cells. (B) Representative plot showing the MAIT cells frequency in patients developing infection or not.

Venous blood samples were collected in sodium heparin tubes and peripheral blood mononuclear cells isolated using Ficoll gradient centrifugation and cryopreserved in 10% Dimethyl Sulfoxide. Batched flow cytometry staining of frozen samples were performed including an even fraction of control and patient samples using the following antibodies for MAIT cell identification: Dead Cell Marker Aqua (Thermofisher, ratio 1:100), anti-CD14-V500 (BD Biosciences, clone: M5E2, ratio 1:100), anti-CD19-V500 (BD Biosciences, clone: HIB19, ratio: 1:100), anti-CD3-BV570 (Biolegend, clone:UCHT1, ratio: 1:50), anti-CD4-BUV615 (BD Biosciences, clone: SK3, ratio: 1:100), anti-CD161-BV650 (BD Biosciences, clone: DX12, ratio: 1:25), and anti-TCR Vα7.2-PE (Biolegend, clone: 3C10, ratio: 1:50). Cells were acquired using a BD FACSymphony A5. After compensation, MAIT cells were defined by first removing dead cells, B cells and monocytes to avoid background and unspecific binding of antibodies. Thereafter, total CD3-expressing cells were identified, CD4-positive cells excluded and MAIT cells defined as CD161 and TCR Vα7.2 double-expressing cells. A gating scheme to identify MAIT cells out of CD3^+^CD4^-^ cells and plots for CD161 and Vα7.2 in individuals with and without bacterial infection is presented in Fig 2.

#### Follow-up

Baseline and follow-up data were collected from chart review. Incident cases of bacterial infection and decompensation events in addition to vital status and cause of death were registered. All patients had at least two years of possible follow-up. Patients were censored at time of death, liver transplantation, emigration or at the end of study period (June 30, 2021).

### Statistical analysis

Continuous data are presented as medians with interquartile ranges (IQR) and categorical variables as total numbers and percentages. To assess any threshold effect, the fraction of MAIT cells out of CD3^+^CD4^-^ cells were divided in quartiles, and patients with the 25% lowest fractions of MAIT cells were used as the reference group. Differences between groups were tested using the Chi-square test or Kruskal-Wallis test as appropriate. A competing risk regression was performed to assess the association between MAIT cell fractions and the cumulative incidence of bacterial infection and hepatic decompensation, respectively. Liver transplant and death from other causes than bacterial infection or decompensation in the respective models were considered competing events. A Cox regression survival analysis was also performed to assess any association between MAIT cells and overall mortality, and the proportional-hazards assumption was verified using Schoenfeld residuals. We also performed a Cox regression to investigate the etiological association between MAIT cells and infections. All models were adjusted for age, sex and severity of liver disease assessed by model of end-stage liver disease (MELD-Na) at the time of inclusion. A sensitivity analysis was performed using a wider definition of infection and another sensitivity analysis excluding the two patients included that were admitted to hospital. Statistical analysis was performed using STATA version 15.1. A two-tailed p-value <0.05 was considered statistically significant.

### Ethical considerations

Written informed consent were obtained from all participants. The study was approved by the regional ethics committee at Karolinska Institutet (Reg nr 2013/2285-31/3).

## Results

We included 106 patients with cirrhosis, median age was 63 years and 64% were men. At inclusion, 104/106 patients were outpatients attending our clinic due to several reasons such as regular visits, refractory ascites, and paracentesis and 2/106 were inpatients admitted to hospital due to decompensating events (one patient with encephalopathy and the other one due to ascites that needed to be drained within a shorter time than the outpatient clinic could arrange). At baseline, 66% of patients had compensated cirrhosis. Most patients, (52/106, 49%) were Child-Pugh A, 36/106 (34%) Child B and 18/106 (17%) were Child C. The most common cause of liver cirrhosis was alcohol-related liver disease (53/106, 50%).

**Fig 3:**
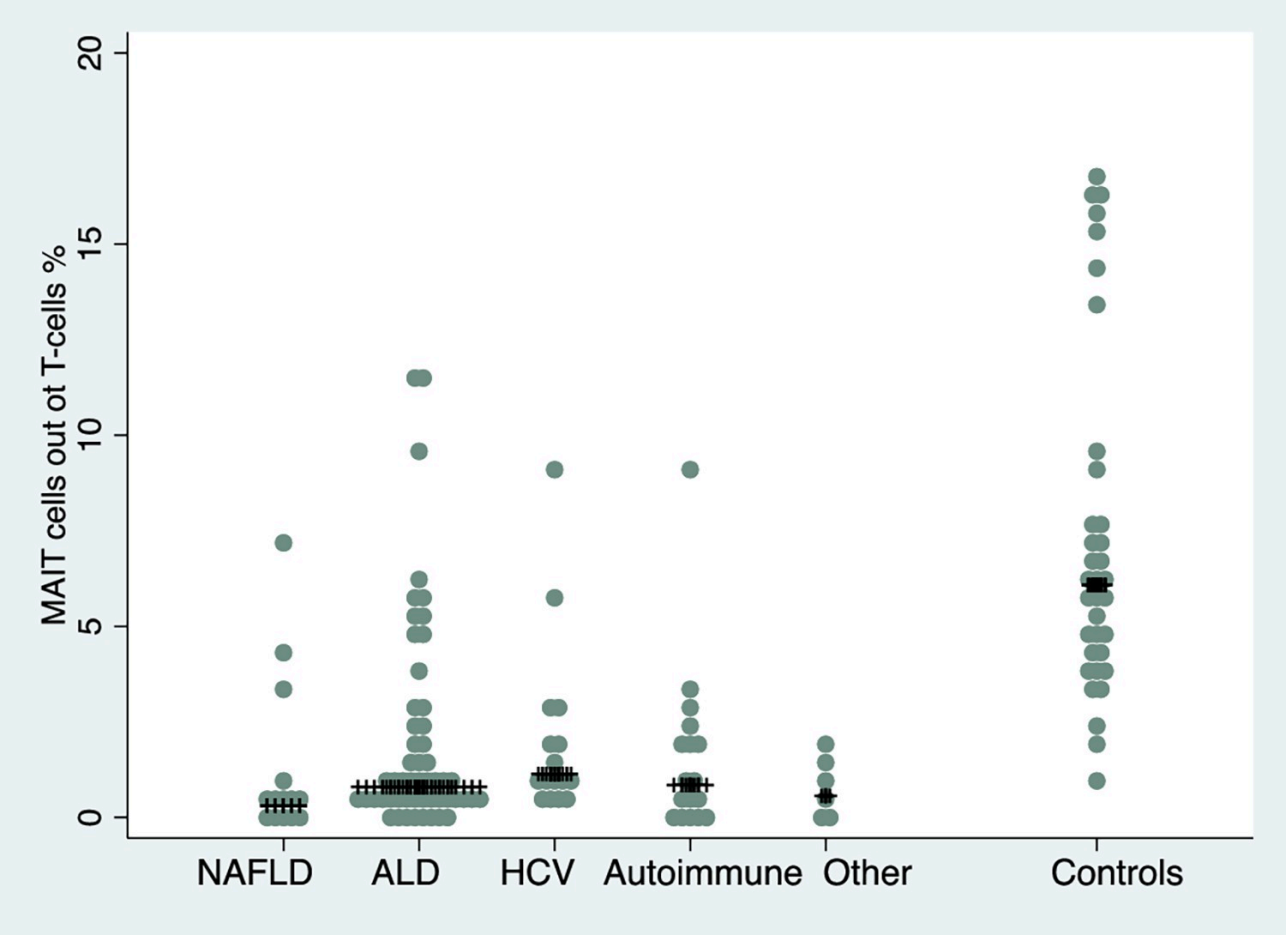
Box plot of median MAIT cell proportions in different liver diseases and controls. No significant difference in median MAIT cell proportions between different liver diseases were seen (p = 0.12).

In healthy individuals, the median MAIT cell fraction was 6.1% out of CD3^+^CD4^-^ cells compared to a median MAIT cell fraction of 0.8% (p<0.01) in all individuals with cirrhosis. No significant difference in the fraction of MAIT cells was seen for different Child-Pugh classes (A = 0.9%, B = 0.6% and C = 0.9%, p = 0.72). Further, there was no difference in MAIT cell levels between patients with and without previous or ongoing decompensation (1.99 vs 1.75% out of T-cells, p = 0.63) or patients with and without immunosuppressants (1.3 vs 0.8% out of T-cells, p = 0.9). The highest median MAIT cell fraction was seen in patients with hepatitis C, but there was no significant difference between disease etiologies (p = 0.12). Levels of MAIT cells stratified on cirrhosis etiology are presented in Fig 3. For every MAIT cell quartile (q), where the first quartile (lowest MAIT cell level) was used as the reference group, the risk for infection increased (adjusted subdistribution hazard ratio (aSHR) for q2 vs q1 1.24 (95%CI 0.43–3.55), for q3 1.41 (95%CI 0.50–3.94) and for q4 1.81 (95%CI 0.65–5.07) indicating a dose-response relationship although this was not statistically significant (p_trend_ = 0.69). Additional baseline characteristics stratified on Child-Pugh class and MAIT cell quartiles are presented in Tables 1 and 2.

**Table 1:** Baseline characteristics for all cirrhosis patients and stratified on Child Pugh class.

Baseline characteristics	All patients with cirrhosis (n = 106)	Child-Pugh A (n = 52)	Child-Pugh B (n = 36)	Child-Pugh C (n = 18)	P-value	Healthy controls (n = 35)
Age (years, median/IQR)	63 (53–70)	66 (60–71)	61 (52–71)	52 (50–57)	<0.01	61
Sex (men, N/%)	68 (64%)	32 (62%)	23 (64%)	13 (72%)	0.80	21 (61%)
MELD-Na (median, IQR)	12 (9–15)	9 (7–12)	14 (11–15)	22 (19–23)	<0.01	
BMI (kg/m^2^, median, (IQR))	27 (24–31)	28 (24–33)	26 (23–31)	26 (23–29)	0.18	
%MAIT cells/T-cells (median/IQR)	0.80 (0.29–2.01)	0.89 (0.33–2.47)	0.64 (0.27–1.85)	0.89 (0.54–1.94)	0.72	6.08 (4.12–9.19)
Compensated at baseline n (%)	71 (67%)	5 (10%)	16 (44%)	15 (83%)	<0.01	
**Cirrhosis etiology n** (% of all patients and of each Child Pugh class)					**0.3**	
NAFLD	14 (13%)	10 (19%)	4 (11%)	0		
Alcohol	53 (50%)	20 (38%)	19 (52%)	14 (78%)		
Hepatitis C	16 (15%)	11 (15%)	5 (14%)	0		
Autoimmune	17 (16%)	9 (17%)	4 (11%)	4 (22%)		
Cryptogenic/other	6 (6%)	2 (4%)	4 (11)	0		
**LAB**						
Bilirubin micromole/L (median/(IQR))	19 (10–39)	11 (8–14)	28 (18–39)	63 (48–145)	<0.01	
PK-INR (median/IQR)	1.2 (1.1–1.4)	1.1 (1.0–1.2)	1.4 (1.2–1.5)	1.7 (1.4–2.0)	<0.01	
Albumin (median/IQR)	32 (26–36)	36 (34–39)	28 (25–32)	23.5 (20–27)	<0.01	
Platelets (median/IQR)	149 (101–195)	166 (111–209)	129 (91–180)	139 (82–166)	0.08	
Leukocytes (median/IQR)	6 (4.4-7-6)	6.0 (4.7–6.9)	5.8 (3.9–7.8)	7.1 (4.9–8.2)	0.46	
Neutrophils (median/IQR)	3.6 (2.3–4.7)	3.6 (2.3–4.6)	3.4 (2.1–4.6)	4.0 (2.8–6.4)	0.18	
**Ascites n** (% of all patients and of each Child Pugh class)					**<0.01**	
No ascites	59 (56%)	45 (86%)	13 (36%)	1 (6%)		
Controlled by diuretics	15 (14%)	2 (4%)	9 (25%)	4 (22%)		
Paracentesis or increased diuretics the 6 past months	26 (25%)	3 (6%)	10 (28%)	13 (72%)		
TIPS	6 (6%)	2 (4%)	4 (11%)	0		
**Encephalopathy n** (% of all patients and of each Child Pugh class)					**<0.01**	
No encephalopathy	96 (91%)	52 (100%)	31 (86%)	13 (72%)		
Mild HE grade 1–2	9 (8%)	0	5 (14%)	4 (22%)		
Severe HE grade 3–4	1 (<1%)	0	0	1 (6%)		
**Varices n** (% of all pat).					**0.11**	
No varices	59 (56%)	35 (67%)	14 (40%)	10 (56%)		
Varices, no bleeding	42 (40%)	16 (31%)	19 (54%)	7 (39%)		
Varices, bleeding within 6 months from baseline	4 (4%)	1 (2%)	2 (6%)	1 (5%)		

Abbreviations: MAIT cells mucosal-associated invariant T-cells, IQR interquartile range, MELD model of end-stage liver disease, NAFLD non-alcoholic fatty liver disease, TIPS trans-jugular intrahepatic portosystemic shunt, BMI body mass index, HE hepatic encephalopathy.

**Table 2:** Baseline characteristics for all cirrhosis patients stratified on MAIT%/T-cells in quartiles.

MAIT cells/T-cells in quartiles	% MAIT cells/T-cells Quartile 1 (n = 27)	% MAIT cells/T-cells Quartile 2 (n = 26)	% MAIT cells/T-cells Quartile 3 (n = 27)	% MAIT cells/T-cells Quartile 4 (n = 26)	P-value
Age median (IQR)	70 (59–73)	63 (52–68)	61 (54–69)	58 (52–68)	0.05
Sex % men	13 (48%)	18 (69%)	19 (70%)	18 (69%)	0.27
MELD-Na median (IQR)	13 (8–17)	13 (10–16)	12 (8–18)	11 (8–14)	0.66
%MAIT/T-cells median (IQR)	0.20 (0.13–0.24)	0.57 (0.41–0.69)	1.17 (0.89–1.87)	4.90 (2.99–6.35)	<0.01
**Cause for cirrhosis (**n, % etiology of each MAIT quartile)					**0.12**
NAFLD	7 (26%)	4 (15%)	0	3 (12%)	
Alcohol	10 (37%)	15 (58%)	13 (48%)	15 (58%)	
Hepatitis C	2 (8%)	4 (16%)	6 (22%)	4 (15%)	
Autoimmune	5 (19%)	3 (12%)	5 (19%)	4 (15%)	
Cryptogenic/other	3 (11%)	0	3 (11%)	0	
**LAB**					
Bilirubin micromole/L median (IQR)	21 (12–39)	22 (13–35)	23 (11–48)	12 (9–35)	0.44
PK-INR (IQR)	1.2 (1.1–1.4)	1.2 (1.1–1.4)	1.2 (1.1–1.5)	1.2 (1.1–1.3)	0.96
Albumin (IQR)	32 (24–34)	31 (26–38)	34 (26–37)	33 (28–36)	0.37
Platelets (IQR)	149 (105–195)	158 (115–204)	144 (100–195)	131 (90–179)	0.74
Leukocytes (IQR)	6.6 (4.9–8.0)	5.8 (4.4–7.0)	5.4 (4.2–7.2)	6.2 (3.7–7.1)	0.57
Neutrophils (IQR)	3.6 (2.1–4.9)	3.6 (2.3–4.8)	3.7 (2.3–4.8)	3.7 (2.3–4.2)	0.95
**Ascites n (% of quartile)**					
No ascites	15 (55%)	17 (65%)	15 (55%)	12 (46%)	
Controlled by diuretics	4 (15%)	2 (8%)	4 (15%)	5 (19%)	
Paracentesis or increased diuretics the past 6 months	6 (22%)	6 (23%)	7 (26%)	7 (27%)	
TIPS	2 (7%)	1 (4%)	1 (4%)	2 (8%)	
**Encephalopathy n (% of quartile)**					**0.24**
No encephalopathy	23 (85%)	23 (88%)	24 (89%)	26 (100%)	
Mild HE grade 1–2	4 (15%)	3 (12%)	2 (7%)	0	
Severe HE grade 3–4	0	0	1 (4%)	0	
**Varices n (% of quartile).**					**0.40**
No varices	15 (58%)	18 (69%)	14 (52%)	12(46%)	
Varices, no bleeding	9 (35%)	7 (27)	13 (48%)	13 (50%)	
Varices, bleeding within 6 months from baseline	2 (7%)	1 (4%)	0	1 (4%)	
**Child-Pugh class n (% of quartile)**					**0.84**
Child-Pugh A	12 (44%)	12 (46%)	14 (52%)	14 (54%)	
Child-Pugh B	12 (44%)	9 (35%)	7 (26%)	8 (31%)	
Child-Pugh C	3 (12%)	5 (19%)	6 (22%)	4 (15%)	

Abbreviations: MAIT cells mucosal-associated invariant T-cells, IQR interquartile range, MELD model of end-stage liver disease, NAFLD non-alcoholic fatty liver disease, TIPS trans jugular intrahepatic portosystemic shunt, BMI body mass index, HE hepatic encephalopathy.

### Risk of infection

During a follow-up of in median 2.5 years (IQR = 0.4–3.8), 32/106 patients (30%) developed an infection. Eleven patients had urinary tract infection, four had spontaneous bacterial peritonitis, four had bacteremia and one pneumonia as the first incident infection. Twelve patients had other infections such as *Clostridioides difficile* infection, cholangitis and skin infections. Interestingly, higher percentages of MAIT cells in blood were associated with a slightly increased risk of bacterial infection in the adjusted analysis (aSHR = 1.15 (95%CI = 1.01–1.31)) but no association was seen in the crude analysis (SHR = 1.10 (95%CI = 0.97–1.25)). Estimates from the crude and adjusted competing risk model stratified on quartiles of MAIT cells are presented in Table 3. Excluding the two patients with acute decompensation at baseline yielded identical results as the main analysis (data not shown).

**Table 3:** Results from competing risk regression investigating association between quartiles of MAIT cells and incident bacterial infection. Death and liver transplantation were considered competing events.

Competing risk regression- *bacterial infection*	Number exposed	Number of events	SHR	95%CI	aSHR	95%CI
% MAIT cells/T-cells	106	32	1.10	0.97–1.25	1.15	1.01–1.31
Age	106	32	1.00	0.97–1.03	1.01	0.97–1.04
MELD-Na	104	30	1.05	0.98–1.12	1.05	0.99–1.12
**Quartile 1**	27	7	reference		reference	
% MAIT cells/T-cells						
**Quartile 2**	26	8	1.05	0.38–2.88	1.16	0.35–3.80
% MAIT cells/T-cells						
**Quartile 3**	27	8	1.17	0.43–3.16	1.46	0.52–4.13
% MAIT cells/T-cells						
**Quartile 4**	26	9	1.51	0.55–4.06	2.25	0.71–7.13
% MAIT cells/T-cells						

Abbreviations: MAIT cells mucosal-associated invariant T-cells, SHR subdistribution hazard ratio, aSHR adjusted subdistribution hazard ratio, CI confidence interval, MELD model of end-stage liver disease, Na sodium.

**Fig 4:**
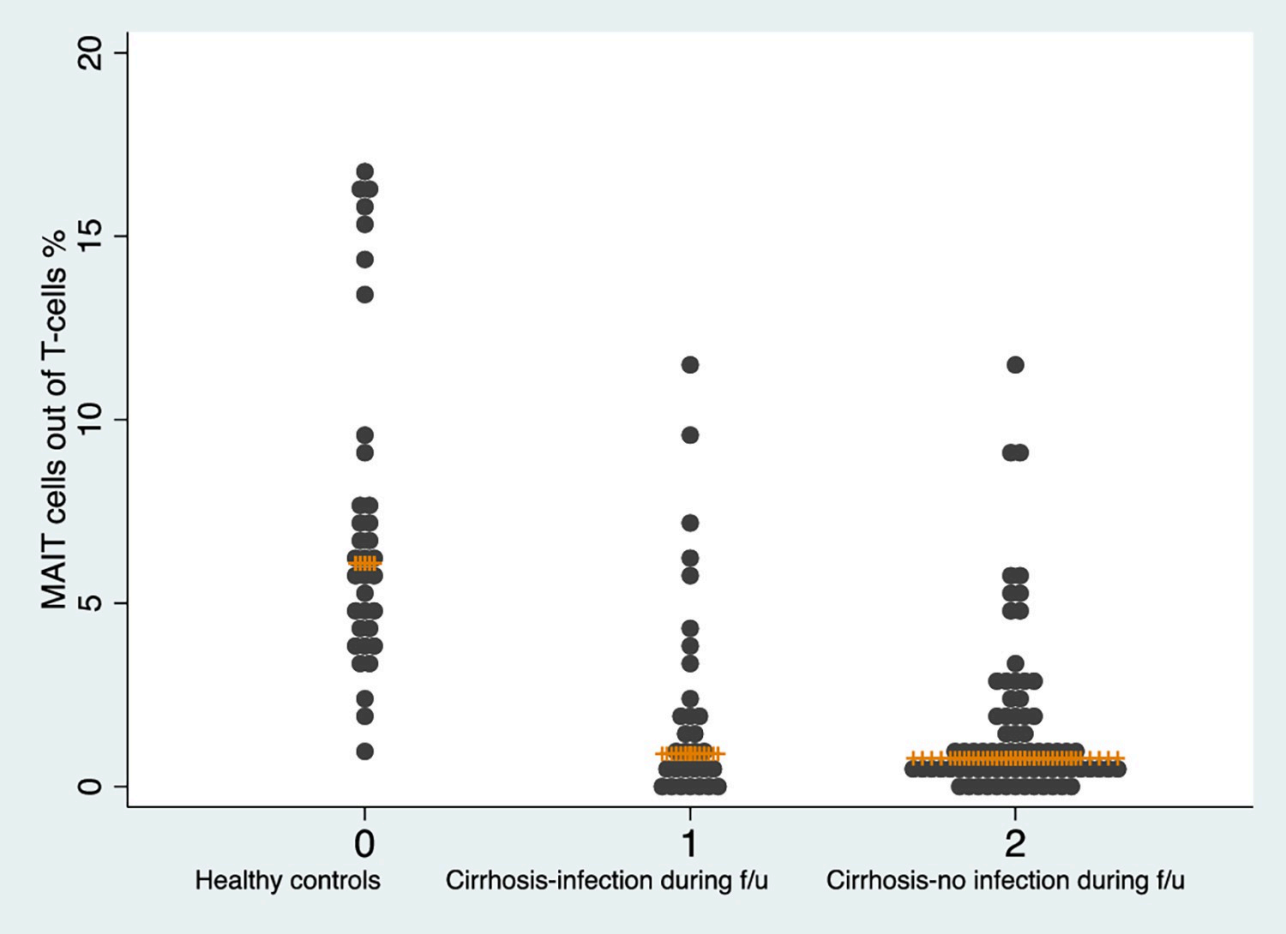
Dot plot of MAIT-cells measured at baseline in healthy controls and in patients with cirrhosis that developed and did not develop an infection during follow-up. The orange line represents the median.

The results from the Cox regression model investigating the association between fraction of MAIT cells and infection yielded almost identical result as the main model (aHR = 1.15, 95%CI = 1.02–1.31). A sensitivity analysis where a wider definition of infection was used, including both viral and bacterial infections diagnosed both in hospitals and primary care, showed similar results (aSHR = 1.17, 95%CI = 1.04–1.31). These data are presented in S1 Table of S1 File. In a post-hoc analysis, we also stratified the cohort on a) sex and b) alcohol-related liver cirrhosis vs. other etiologies. We found an association between MAIT cell levels and incident infections in men, but no significant results were found in women. This finding should be interpreted with some caution given the limited sample size with a low proportion of women (n = 38, 36%). There were no indications of a difference in risk of bacterial infections based on etiology. These estimates are presented in S2 Table of S1 File. A dot plot showing the individual values of MAIT-cell levels for healthy controls and patients with cirrhosis who develop and not develop infection is presented in Fig 4.

### Risk of decompensating event and death

During follow-up, 11 of 71 (15%) patients free of decompensation at baseline developed decompensation. Higher MAIT cell percentage was not associated with a higher risk of hepatic decompensation (aSHR = 1.19, 95%CI = 0.91–1.56) or with risk of death (adjusted hazard ratio (aHR) 1.10 (95%CI = 0.97–1.22)). In Table 4, estimates for the risk of hepatic decompensation are presented, and in Table 5 the estimates for overall mortality are presented. A survival curve of overall mortality is presented in S1 Fig.

**Table 4:** Results from competing risk regression investigating association between quartiles of MAIT cells and incident hepatic decompensation. Death and liver transplantation were considered competing events.

Competing risk regression-*decompensation*	Number exposed	Number of events	SHR	95%CI	aSHR	95%CI
% MAIT cells/T-cells	71	11	1.12	0.83–1.52	1.19	0.91–1.56
Age	71	11	0.99	0.96–1.03	1.01	0.97–1.06
MELD-Na	69	9	1.08	0.93–1.27	1.09	0.92–1.30
**Quartile 1**	18	3	reference		reference	
% MAIT cells/T-cells						
**Quartile 2**	18	4	1.41	0.31–6.51	1.45	0.21–10.19
%MAIT cells/T-cells						
**Quartile 3**	19	2	0.57	0.10–3.24	0.60	0.09–4.03
% MAIT cells/T-cells						
**Quartile 4**	16	2	0.72	0.12–4.38	1.21	0.14–10.43
%MAIT cells/T-cells						

Abbreviations: MAIT cells mucosal-associated invariant T-cells, SHR subdistribution hazard ratio, aSHR adjusted subdistribution hazard ratio, CI confidence interval, MELD model of end-stage liver disease, Na sodium.

**Table 5:** Results from Cox regression investigating association between quartiles of MAIT cells and incident overall mortality.

Cox regression–*overall mortality*	Number exposed	Number of events	HR	95%CI	aHR	95%CI
% MAIT cells/T-cells	106	26	1.08	0.95–1.22	1.08	0.95–1.22
**Age**	106	26	1.00	0.97–1.04	1.04	0.99–1.09
**MELD-Na**	104	24	1.08	1.01–1.15	1.12	1.03–1.22
**Quartile 1**	27	7	reference		reference	
% MAIT cells/T-cells						
**Quartile 2**	26	8	1.33	0.48–3.68	1.38	0.44–4.32
%MAIT cells/T-cells						
**Quartile 3**	27	5	0.70	0.22–2.21	0.71	0.21–2.38
% MAIT cells/T-cells						
**Quartile 4**	26	6	0.80	0.27–2.39	0.92	0.28–3.01
%MAIT cells/T-cells						

Abbreviations: MAIT cells mucosal-associated invariant T-cells, HR hazard ratio, aHR adjusted hazard ratio, CI confidence interval, MELD model of end-stage liver disease, Na sodium.

## Discussion

In this study we found that in the context of MAIT cell loss in patients with cirrhosis, relatively preserved MAIT cell frequency in peripheral blood was associated with a slightly higher risk of bacterial infection. Further, we did not find a significant association between frequencies of MAIT cells and decompensation or death, although the point estimates of all analyses were in the same direction. Several previous studies [17–22] report diminished numbers of MAIT cells in the circulation in patients with cirrhosis compared to healthy controls and the cell phenotype is often hyperactivated and exhausted. One hypothesis is that a continuous stimulation leads to apoptotic cells and diminished number of MAIT cells, and another hypothesis is that MAIT cells are recruited to the inflamed liver with consequently lower MAIT levels in peripheral blood [23]. The significance of lower MAIT cell fractions on longitudinal outcomes such as bacterial infections have not been determined [24]. As opposed to our hypothesis, we found that in patients with cirrhosis, MAIT cell levels closer to levels found in healthy controls was associated with a higher risk to develop bacterial infections. Another explanation could be residual confounding, in that patients with higher levels of MAIT-cells are sicker than other patients. Although we adjusted for the main risk factors of infections, the limited number of outcomes precludes a more detailed analysis of this.

In line with recent research [25] we report the median fraction of MAIT cells to be less than one percent of CD3^+^CD4^-^ cells in patients with cirrhosis. Further, we found that in cirrhosis, MAIT cell percentages were lower in older individuals, which has also been shown in healthy individuals [26, 27].

There is inconclusive evidence regarding MAIT cell levels and liver disease severity from cross-sectional studies. Hegde et al report no association between MAIT cell levels and liver disease severity in a study of 74 patients with biopsy-proven cirrhosis [25]. Niehaus et al found similar frequencies of peripheral MAIT cells in patients with compensated and decompensated cirrhosis [17] and von Seth et al found no correlation to disease severity when investigating MAIT cells in patients with primary sclerosing cholangitis [28]. In contrast, Zhang et al, reported a correlation of MAIT cell frequency and liver disease severity estimated with laboratory tests [29]. Here, we found no correlation between levels of peripheral blood MAIT cells and liver cirrhosis severity at baseline, or on the risk of future decompensation. Thus, our study supports the notion that MAIT cells in blood do not seem to be correlated with liver disease severity, although larger studies are needed to confirm this.

Strengths of this study include the prospective cohort design entailing less risk of bias than previous cross-sectional studies in this research field. The long follow-up is another strength. Outcomes were ascertained by thorough chart review, increasing internal validity. Finally, the MAIT cell percentage was determined using multi-color flow cytometry and experiments performed on cryopreserved samples in a batch setup allowing for robust results.

One limitation with our study is that the electronic patient chart used cover seven out of eight hospitals in the Stockholm region, but not hospitals outside the Stockholm region, why events occurring outside this setting could not be captured. However, all patients resided in the Stockholm region, and we believe this is unlikely to affect our results. Another consideration is that the level of MAIT-cells might not reflect long-term infection risk although some evidence exist that MAIT cells remain unchanged over time. Hengst et al obtained blood samples from patients with HCV before, during and after successful HCV treatment. 77% of the patients had cirrhosis and the virus was cleared in 8 weeks. Throughout the follow-up period of 72 weeks MAIT cells did not return to levels found in healthy individuals but remained decreased [30]. Future studies should include larger cohorts to ensure enough short-term infection outcomes, and repeated sampling of MAIT-cells would be interesting to investigate. A potential bias is that patients treated more often in hospital, including patients with more severe liver disease but also patients that suffer from other diseases, might be diagnosed with more infections in hospital, as they are more carefully followed than less sick patients. Severity of liver disease was adjusted for in the regression models, but the low sample size did not allow for adjustment of other possible confounders. Furthermore, despite the long follow-up period, there were relatively few outcomes, which results in more uncertain estimates and did not allow for a more granular analysis, for instance on subtypes of infections or clinically relevant subgroups such as sex. Finally, we were not able to study phenotypical and functional aspects of the MAIT cell compartment that may influence their effectiveness against bacterial infections.

### Implications

Our findings do not distinctly support that MAIT cells in peripheral blood can be used to assess risk of future infection in patients with cirrhosis. This needs to be further investigated in studies with a larger sample size than here for additional investigation of potential cofounders. Future studies should also evaluate the association between MAIT-cell levels and other outcomes, such as acute on chronic liver failure. It is also warranted to gain a better understanding of the basic immunobiology of MAIT cells in pre-clinical settings to be able to explain the pathophysiological mechanisms for the association between relatively higher levels of MAIT cells and risk of infections.

## Conclusion

In patients with cirrhosis, relatively preserved MAIT cell levels in peripheral blood were associated with a higher risk to develop bacterial infections. These findings must be further investigated in a larger setting to be able to draw any conclusions about the capacity of MAIT cells to predict infections in patients with cirrhosis.

## Supporting information

S1 Fig Kaplan-Meier survival curve stratified on MAIT-cell quartiles. (TIF)

S1 File (DOCX)

## Abbreviations

MAIT cells: mucosal-associated invariant T-cells
aSHR: adjusted subdistribution hazard ratio
aHR: adjusted hazard ratio
HCC: hepatocellular carcinoma
HIV: human immunodeficiency virus
MELD: model of end-stage liver disease
Na: sodium
IQR: interquartile ranges
BMI: body mass index
CI: confidence interval
NAFLD: non-alcoholic fatty liver disease
TIPS: trans jugular intrahepatic portosystemic shunt
HE: hepatic encephalopathy
