# Supplementary figures and images for: Evaluation of mucosal-associated invariant T-cells as a potential biomarker to predict infection risk in liver cirrhosis
Source: PLoS One. 2024 May 1;19(5):e0294695. doi: 10.1371/journal.pone.0294695 (PMC11062522; doi:10.1371/journal.pone.0294695)

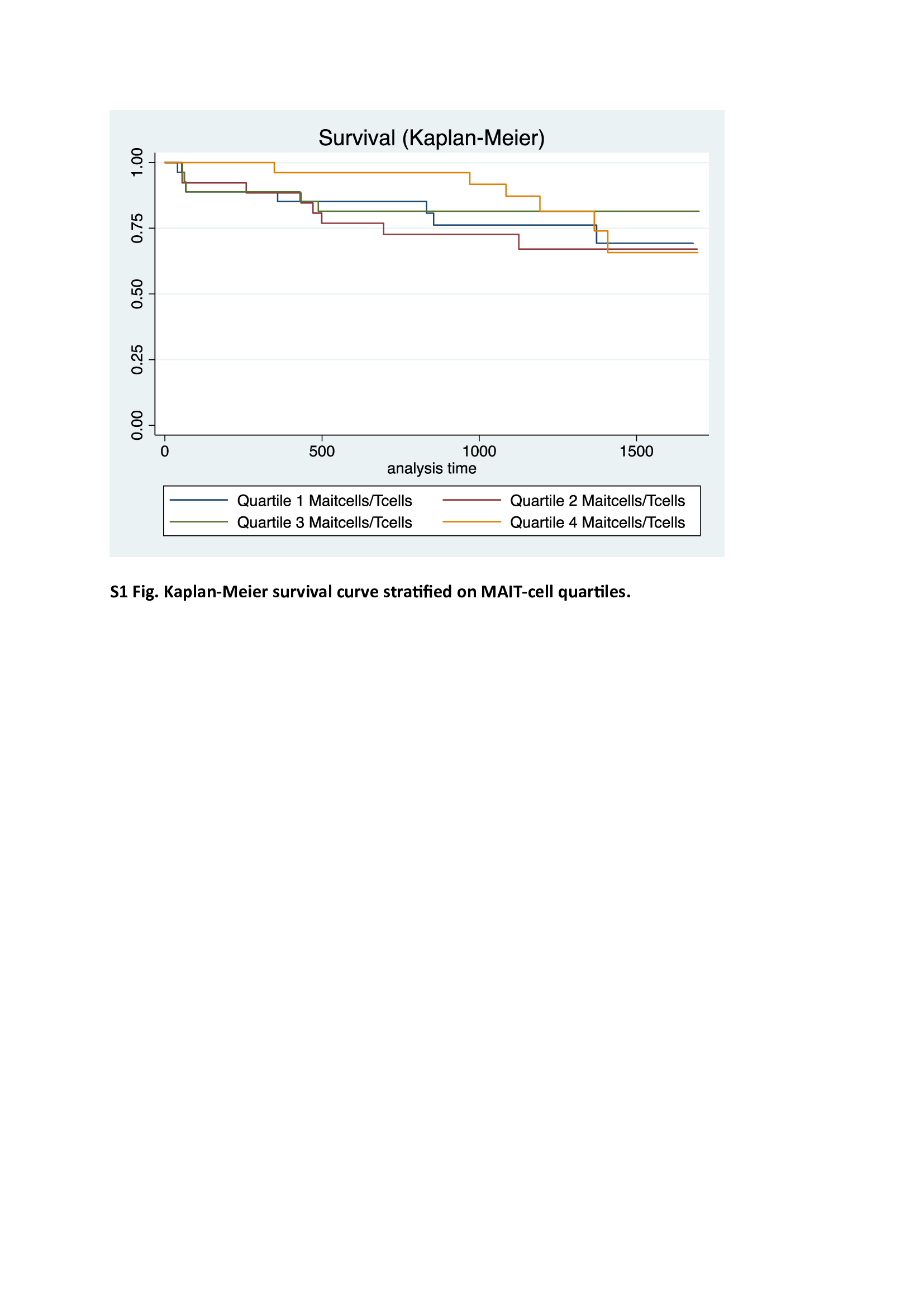

Supplement: S1 Fig — (TIF) [file pone.0294695.s001.tif]
