# Supplementary material for: Evaluation of mucosal-associated invariant T-cells as a potential biomarker to predict infection risk in liver cirrhosis
Source: PLoS One. 2024 May 1;19(5):e0294695. doi: 10.1371/journal.pone.0294695 (PMC11062522; doi:10.1371/journal.pone.0294695)
Supplement: S1 File — (DOCX) [file pone.0294695.s002.docx]

**Evaluation of Mucosal-associated invariant T-cells as a potential biomarker to predict infection risk in liver cirrhosis-** Supplementary data**.**

**Definitions**

**Infection**

Defined as a bacterial infection that was diagnosed in a hospital (out-clinic visit) or during hospitalization and required treatment with antibiotics.

1) spontaneous bacterial peritonitis (defined by ascitic fluid neutrophilic count >0.25x10^9^/l),

2) urinary tract infection (requiring a positive culture),

3) respiratory tract infection (chest x-ray consistent with pneumonia in combination with typical symptoms),

4) bacteremia (defined as a positive blood culture without source of infection identified)

5) others (clinically relevant such as endocarditis, wound infection, meningitis, and gastrointestinal tract infection). A positive culture from affected site of infection was required.

**Decompensation event**

Patients were defined decompensated at baseline:

Ascites and paracentesis up to three months before baseline, or new anti-diuretic medication or increased dosage three months from baseline. Patients with encephalopathy grade 3-4 and bleeding varices up to six months from baseline were also considered as decompensated at baseline. Patients with TIPS were also considered decompensated at baseline.

A decompensation event during follow-up:

Defined as ascites and paracentesis or new anti-diuretic medication or increased dosage, encephalopathy grade 3-4 and/or bleeding varices.

**Bleeding varices**

Endoscopically verified or GI-bleeding in a patient with known varices

| **Sensitivity analysis- wider definition infection** | **Number exposed** | **Number of events** | **SHR** | **95%CI** | **aSHR** | **95%CI** |
| --- | --- | --- | --- | --- | --- | --- |
| % MAIT cells /T-cells | 106 | 41 | 1.13 | 1.00-1.26 | 1.17 | 1.04-1.31 |
| **Quartile 1**  % MAIT cells/T-cells | 27 | 7 | reference |  | reference |  |
| **Quartile 2**  % MAIT cells/T-cells | 26 | 11 | 1.80 | 0.72-4.49 | 2.25 | 0.80-6.34 |
| **Quartile 3**  % MAIT cells/T-cells | 27 | 11 | 1.76 | 0.69-4.48 | 2.34 | 0.85-6.42 |
| **Quartile 4**  % MAIT cells/T-cells | 26 | 12 | 2.24 | 0.88-5.71 | 3.38 | 1.17-9.75 |

**Table S1. Sensitivity analysis. Competing risk regression bacterial infection, wider definition.** Abbreviations: SHR subdistribution hazard ratio, MAIT cells mucosal-associated invariant T-cells, aSHR adjusted subdistribution hazard ratio, CI confidence interval

| **Competing risk regression- *bacterial infection*** | **Number exposed** | **Number of events** | **SHR** | **95%CI** | **aSHR** | **95%CI** |
| --- | --- | --- | --- | --- | --- | --- |
| **Sex** |  |  |  |  |  |  |
| Men | 68 (64%) | 19 (28%) | 1.14 | 1.01-1.29 | 1.21 | 1.07-1.37 |
| Women | 38 (36%) | 12 (32%) | 0.82 | 0.51-1.32 | 0.48 | 0.17-1.37 |
| **Etiology** |  |  |  |  |  |  |
| ALD | 53 (50%) | 18 (34%) | 1.11 | 0.96-1.29 | 1.15 | 0.97-1.36 |
| Non-ALD | 53 (50%) | 13 (25%) | 1.04 | 0.80-1.35 | 1.13 | 0.85-1.49 |

**Table S2. Competing risk regression of incident bacterial infection stratified on sex and alcohol-related liver cirrhosis vs. other etiologies.** Abbreviations: ALD alcohol-related liver disease, SHR subdistribution hazard ratio, MAIT cells mucosal-associated invariant T-cells, aSHR adjusted subdistribution hazard ratio, CI confidence interval

**Fig. S1 Kaplan-Meier survival curve stratified on MAIT-cell quartiles.**

**Levels of MAITcells/Tcells in each study participant**

0.77

0.22

7.42

0.63

0.23

0.80

0.05

9.50

0.34

0.54

0.19

0.89

0.23

2.44

4.19

0.25

1.56

0.83

1.45

0.77

0.24

3.95

5.68

0.14

8.89

2.48

0.34

0.71

1.11

0.24

0.11

1.49

0.16

3.06

0.21

0.23

6.35

0.39

2.98

5.78

0.49

0.63

0.52

0.05

0.11

5.43

1.56

0.48

0.79

1.01

5.78

0.28

4.91

1.88

0.68

0.85

0.80

0.13

0.98

9.02

0.66

0.20

1.91

5.33

0.62

0.48

2.99

0.85

0.41

2.81

0.69

2.45

0.18

11.50

11.30

0.39

0.13

0.93

1.87

0.71

1.05

1.26

0.29

1.98

0.27

1.02

0.33

3.26

2.01

0.14

0.44

0.04

0.33

4.89

1.69

1.17

0.78

2.92

3.29

0.27

0.15

0.22

0.60

0.85

1.94

1.91
